# Development and In-Depth Characterization of Bacteria Repellent and Bacteria Adhesive Antibody-Coated Surfaces Using Optical Waveguide Biosensing

**DOI:** 10.3390/bios12020056

**Published:** 2022-01-20

**Authors:** Eniko Farkas, Robert Tarr, Tamás Gerecsei, Andras Saftics, Kinga Dóra Kovács, Balazs Stercz, Judit Domokos, Beatrix Peter, Sandor Kurunczi, Inna Szekacs, Attila Bonyár, Anita Bányai, Péter Fürjes, Szilvia Ruszkai-Szaniszló, Máté Varga, Barnabás Szabó, Eszter Ostorházi, Dóra Szabó, Robert Horvath

**Affiliations:** 1 Centre for Energy Research, Nanobiosensorics Laboratory, Institute of Technical Physics and Materials Science, 1121 Budapest, Hungary; farkas.eniko@ek-cer.hu (E.F.); tarrr@edu.bme.hu (R.T.); tamas.gerecsei@cytosurge.com (T.G.); asaftics@coh.org (A.S.); kovacs.kinga.dora@energia.mta.hu (K.D.K.); peter.beatrix@energia.mta.hu (B.P.); kurunczi.sandor@energia.mta.hu (S.K.); szekacs.inna@ek-cer.hu (I.S.); 2 Department of Electronics Technology, Faculty of Electrical Engineering and Informatics, Budapest University of Technology and Economics, 1111 Budapest, Hungary; bonyar@ett.bme.hu; 3 Department of Biological Physics, Eötvös Loránd University, 1117 Budapest, Hungary; 4 Institute of Medical Microbiology, Semmelweis University, 1089 Budapest, Hungary; stercz.balazs@med.semmelweis-univ.hu (B.S.); domokos.judit@med.semmelweis-univ.hu (J.D.); ostorhazi.eszter@med.semmelweis-univ.hu (E.O.); szabo.dora@med.semmelweis-univ.hu (D.S.); 5 Centre for Energy Research, Microsystems Lab, Institute of Technical Physics and Materials Science, 1121 Budapest, Hungary; banyai.anita@ek-cer.hu (A.B.); furjes.peter@energia.mta.hu (P.F.); 6 77 Elektronika Ltd., 1116 Budapest, Hungary; szszani@e77.hu (S.R.-S.); mvarga@e77.hu (M.V.); bszabo@e77.hu (B.S.)

**Keywords:** bacteria sensing, bacteria repellent coatings, affinity layers, layer structure, binding kinetics, *Escherichia coli*, ELISA, BSA, I-block, PLL-*g*-PEG, PAcrAM-*g*-(PMOXA, NH_2_, Si), polyclonal and monoclonal antibodies (Abs), antibody orientation, OWLS, waveguide sensing, physisorption, Avidin-biotin, AnteoBind, protein A

## Abstract

Bacteria repellent surfaces and antibody-based coatings for bacterial assays have shown a growing demand in the field of biosensors, and have crucial importance in the design of biomedical devices. However, in-depth investigations and comparisons of possible solutions are still missing. The optical waveguide lightmode spectroscopy (OWLS) technique offers label-free, non-invasive, in situ characterization of protein and bacterial adsorption. Moreover, it has excellent flexibility for testing various surface coatings. Here, we describe an OWLS-based method supporting the development of bacteria repellent surfaces and characterize the layer structures and affinities of different antibody-based coatings for bacterial assays. In order to test nonspecific binding blocking agents against bacteria, OWLS chips were coated with bovine serum albumin (BSA), I-block, PAcrAM-*g*-(PMOXA, NH_2_, Si), (PAcrAM-P) and PLL-*g*-PEG (PP) (with different coating temperatures), and subsequent *Escherichia coli* adhesion was monitored. We found that the best performing blocking agents could inhibit bacterial adhesion from samples with bacteria concentrations of up to 10^7^ cells/mL. Various immobilization methods were applied to graft a wide range of selected antibodies onto the biosensor’s surface. Simple physisorption, Mix&Go (AnteoBind) (MG) films, covalently immobilized protein A and avidin–biotin based surface chemistries were all fabricated and tested. The surface adsorbed mass densities of deposited antibodies were determined, and the biosensor;s kinetic data were evaluated to divine the possible orientations of the bacteria-capturing antibodies and determine the rate constants and footprints of the binding events. The development of affinity layers was supported by enzyme-linked immunosorbent assay (ELISA) measurements in order to test the bacteria binding capabilities of the antibodies. The best performance in the biosensor measurements was achieved by employing a polyclonal antibody in combination with protein A-based immobilization and PAcrAM-P blocking of nonspecific binding. Using this setting, a surface sensitivity of 70 cells/mm^2^ was demonstrated.

## 1. Introduction

Optical waveguide lightmode spectroscopy (OWLS) is a surface-sensitive biosensor for probing the surface adhesion and binding of biological and chemical species in a real-time and label-free manner. OWLS detects refractive index changes 100–200 nm above the sensor’s surface, providing quantitative information on near-surface kinetic and structural processes [1,2].

Bacteria repellent surfaces and antibody (Ab)-based coatings for bacterial assays have crucial importance in the field of biosensors, and are highly relevant in the design and development of novel biomedical devices [3,4,5,6]. However, in-depth investigations and comparisons of possible surface coating solutions for this specific application are still missing.

Nowadays there is a rapidly growing demand to develop bacteria repellent coatings. Bacteria can often adhere to surfaces, where they can subsequently form biofilms [7]. In many cases, both in everyday and industrial settings, impeding the adhesion and subsequent biofilm formation of bacteria is essential [8]. Generally, bacterium repellent surfaces prevent the bacterial adhesion step, and can be used for biosensors. Several such antifouling coatings have been developed to inhibit bacterial adhesion, exploiting hydrophobicity, electrostatic interactions, roughness and topographical patterning [9]. These surfaces can be analyzed by biological (polymerase chain reaction; colony formation assay), chemical (extracellular polymeric substances extraction, microtiter plate dye staining and phospholipid-based analysis), physical (ultrasonic time-domain reflectometry, electrochemical impedance spectroscopy (EIS) or electric cell–substrate impedance sensing (ECIS) and weight measure) [10,11] and microscopy (atomic force microscopy, light microscopy and scanning electron microscopy) techniques [12]. From among the large variety of repellent coatings, we focused on two traditional coatings, namely, bovine serum albumin (BSA) and I-block, and two novel polymer coatings, PLL-*g*-PEG (PP) (PLL is poly(L-lysine)-*graft*-poly(ethylene glycol)) and PAcrAM-*g*-(PMOXA, NH_2_, Si) (PAcrAM-P) (PA is poly(acrylamide)-*graft*-poly(2-methyl-2-oxazoline), amine, silane). BSA has been used traditionally in assays as a surface coating to reduce nonspecific binding [13,14,15]. I-block is another conventional reagent containing purified casein that is used against nonspecific adsorption in enzyme-linked immunosorbent assays (ELISA) and other assays [16,17,18]. Among the various polymer layers, PEG is a well-known protein repellent [19,20]. The protein and bacteria repellent properties of PEG result from the hydrophilic, uncharged PEG chains, which freely float in the aqueous phase and form comb-like structures [21]. There are different types of methods for grafting substrates. One elegant way is electrostatically grafting PLL-*g*-PEG through the positively charged amino-groups of poly-L-lysine to negatively-charged metal oxide surfaces [22]. Poly(2-oxazoline)s have been increasingly studied during the last decade as a suitable alternative to PEGs in applications of biomaterials [23]. The novel poly(2-oxazoline)-based coating (PAcrAM-P) was devised with a dimethylsilanol moiety for surface grafting, and with PMOXA side chains, which are responsible for its protein repellency. It was shown that the fabricated patterns of PAcrAM resist outgrowth of the neuronal cells with better efficiency than PEG-based polymers [24]. However, an in-depth comparison of these surfaces for bacteria repellent applications is not available in the literature.

The characterization of Abs is an essential task for the design of biosensors and lab-on-a-chip devices. Immunoglobulin (IgG) Abs are approximately 150 kDa molecular mass proteins which can be divided into fragment antigen-binding (Fab) and fragment crystallizable (Fc) regions. The role of the Fab region is the recognition and binding of the antigen; therefore, an oriented IgG layer with a protruding Fab region is desired for the assay. Well-orientated Abs are found in an “end-on” position, whereas randomly immobilized ones assume “head-on,” “side-on” and “lying-on” arrangements [25]. Various Ab immobilization techniques have been developed on a wide range of surfaces, such as on plasma-treated polymer surfaces, three-dimensional substrates, self-assembled monolayers and molecular imprinted polymers [26,27]. Using many of these strategies, Ab immobilization occurs by physical adsorption to a substrate with increased hydrophobicity, but this method cannot guarantee the proper immobilization of Abs. Another popular immobilization approach is the covalent binding of Ab molecules, assuring oriented and dense binding. The covalent capturing of Abs can be realized through a number of chemical systems. The chemistry applied depends on the substrate functionality, the Ab, the environmental pH and temperature and the degree of conjugation. Most of the covalent binding strategies target amine, carboxyl or thiol groups, or carbohydrate moieties. However, the covalent binding of Abs can change the molecular conformation of the Ab. Affinity immobilization is an alternative technique which may offer a solution to this problem. Affinity immobilization is generally realized by applying binding peptides or proteins (e.g., protein A and G), nucleotide-binding (e.g., DNA-directed immobilization and aptamers) or metal affinity [25,28].

An important field of application where bacteria repellent surfaces play an essential role is biosensors. In the field of biosensors, bacterial detection has been summarized in a recent book [29]. The sensitivity of the immunoassay still remains a limitation, and it depends largely on the actual sensing scheme, along with the assay’s parameters. Recently, there appeared a few reports showing detection limits down to 10^3^ CFU/mL and even lower [30]. The sensitivity issue is even more critical for label-free optical sensors if the whole organism needs to be detected. The limits of detection (LODs) are usually achieved by applying a specifically devised sensing method combined with an efficient surface chemistry to suppress nonspecific signals; it may induce signal amplification [31,32]. This OWLS biosensor can characterize different surfaces, making it an ideal tool for screening bacteria repellent coatings or characterizing receptor molecules, such as Abs, regarding their biorecognition efficiency and selectivity. These fields are essential in biosensor assay development for bacteria. An in-depth comparison of various surfaces for bacteria monitoring is still missing.

In this study, our goal was to develop a method for screening a wide range of bacteria repellent surfaces and characterize the binding performance and affinity of various Ab-based coatings for online label-free monitoring of bacteria. The bacteria repelling abilities of BSA, I-block, PLL-*g*-PEG and PAcrAM-P layers were investigated by real-time OWLS measurements. Different Ab molecules were also immobilized on the OWLS chip surface to detect *Escherichia coli* (*E. coli*) as a case study. For Ab immobilization, we applied various methods, including simple physisorption, immobilization to Mix & Go (AnteoBind) (MG), protein A and avidin–biotin-based layers. The surface mass densities of immobilized Abs were determined in real-time OWLS measurements, and the kinetics of deposition and the structure of formed layers were evaluated in detail. The development of the biosensing affinity layers was supported by ELISA measurements on the binding capabilities of the bacteria-specific Abs. The best performing layer combinations were identified, and the possible limits of detections using these coatings are discussed.

## 2. Materials and Methods

### 2.1. OWLS Instrument and Related Protocols

**Figure 1:**
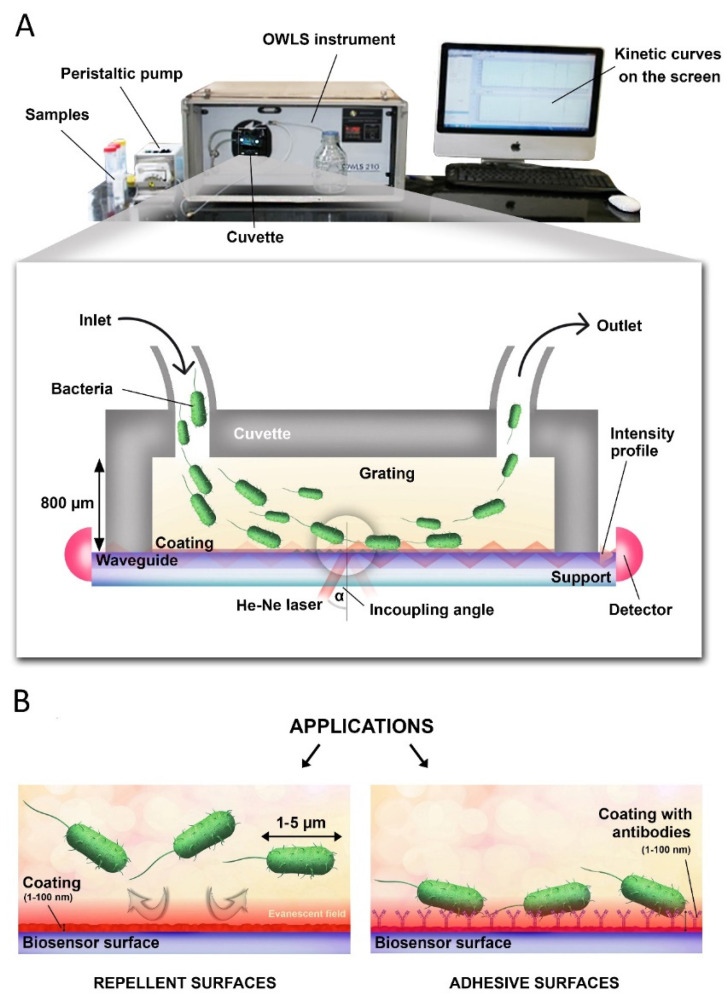
(**A**) Cross-sectional view of the OWLS cuvette and the basics of optical detection. Laser light is coupled into an optical waveguide layer by a surface grating where it propagates by total internal reflection to a photodetector placed at the end of the waveguide. Adsorbing bacteria shift the resonant angle (α). (**B**) OWLS is an ideal tool for testing and developing both bacteria repellent and bacteria adhesive surfaces.

An OWLS210 instrument (MicroVacuum Ltd., Budapest, Hungary) was used to monitor the bacterial adhesion on Ab immobilized surfaces. OWLS is a surface-sensitive technique utilizing a planar optical waveguide chip. This waveguide chip consists of an 8 mm × 16 mm glass substrate on which a TiO_2_-SiO_2_ waveguide layer was made by sol-gel technique (OW2400 sensor chip, MicroVacuum Ltd.), and a 1 mm wide optical grating was formed in this waveguide layer in the center of the chip. The grating on the optical chip is illuminated with a He-Ne laser beam with a wavelength of 632.8 nm. By coupling the laser beam through the grating, an evanescence electromagnetic field is generated, which can probe the changes in refractive index near the optical waveguide surface (within a penetration depth of around 100–200 nm) [1]. In the applied sensor chips, the 0th order transverse electric (TE_0_) and transverse magnetic (TM_0_) waveguide modes can be supported. The OWLS biosensor uses a detector (photodiode) located at both ends of the sensor chip to measure the intensities of light coupled into the waveguide while changing the angle of incidence of the illuminating laser beam. From the measured coupling angles, the effective refractive index values can be calculated using the grating equation (see Figure 1) [2]. From the measured values the adlayer refractive index and thickness can be calculated, from which the adsorbed surface mass density was determined using the de Feijter’s formula [2,33]. The set sampling time was 13 s. During the measurement, the solutions are applied directly to the surface of the waveguide sensor using a liquid cuvette through the injection port (septum) on the top of the cuvette. Liquids are also flowed into the cuvette using a peristaltic pump at a rate of 1 µL/s [34].

Prior to each measurement, the OWLS sensor chips were thoroughly cleaned. The chips were soaked in chromosulfuric acid for 3 s and then rinsed with Milli-Q (ultrapure water) water. Then they were dipped in 0.5 M KOH solution, followed by a Milli-Q water rinse. The chips were sonicated eight times for 5 min in Milli-Q water, and after each cycle the water was changed over the chips, and then the cleaned sensors were dried with nitrogen flow. The dry chips were let stand overnight in the buffer (used for baseline measurements) until the next day measurement.

### 2.2. Blocking Solutions in OWLS Experiments

Blocking of nonspecific interactions was tested by bovine serum albumin (BSA) (Sigma-Aldrich Chemie GmbH, Darmstadt, Germany, catalog number: A8806), I-block (Tropix I-block, Applied Biosystems, Waltman, MA, USA, catalog number: T2015), Poly-(L-lysine)-*graft*-poly(ethylene-glycol) (PLL-*g*-PEG, SuSoS AG, Dübendorf, Switzerland, shortened as PP later on), poly(acryl-amide)-*g*-(PMOXA, 1,6-hexanediamine, 3-aminopropyldimethylethoxysilane) (PAcrAM-*graft*-(PMOXA, NH_2_, Si), SuSoS AG, Dübendorf, Switzerland, shortened as Pacram-P thereafter), which were dissolved in MES buffer, PBS (Sigma-Aldrich Chemie GmbH, Darmstadt, Germany, catalog number: P4417), 10 mM and 1 mM 2-[4-(2-hydroxyethyl)piperazin-1-yl]ethanesulfonic acid (HEPES) buffer, respectively. 

### 2.3. Antibody Immobilization Strategies and Reagents in OWLS Experiments

The Abs were immobilized on three types of coatings prepared on the OWLS sensor chip surface prior to Ab deposition. The reagents used for coating preparation were the following. Mix&Go Biosensor coating agent (shortened as MG from now on) (Anteo Technologies Pty Ltd., Brisbane, Australia, A-PLSCO10) (noteL its new name is AnteoBind), was used for preparing the MG Ab immobilization layer.

GOPS ((3-glycidyloxypropyl)-triethoxysilane, Sigma-Aldrich Chemie GmbH, Darmstadt, Germany, catalog number: 50059) was used to silanize the chip surface for capturing a layer of protein A (from *Staphylococcus aureus*, Sigma-Aldrich Chemie GmbH, Darmstadt, Germany, catalog number: 539202). 

In the case of the avidin–biotin method, the OWLS chip surface was coated with poly-(L-lysine)-*graft*-poly(ethylene-glycol)-20% biotin (PLL-*g*-PEG-20% biotin, SuSoS AG, Dübendorf, Switzerland, shortened as PP-b from now on) to which avidin (VWR, A2568.0010) was bound. This was followed by the addition of the biotinylated Abs (see Ab specification earlier). 

### 2.4. Bacterial Culture

*Escherichia coli* (*E.coli*) DH5α bacteria (ATCC, Manassas, VA, USA, catalog number: 68233) (4 McFarland, 10^9^–10^10^ cells/mL range) diluted in phosphate buffered saline (PBS) in 10^5^–10^9^ cells/mL range and *Staphylococcus epidermidis (S. epidermidis)* bacteria (ATCC, Manassas, VA, USA, catalog number: 14990) (4 McFarland, 10^9^–10^10^ cells/mL range) diluted in PBS or physiological saline solution (0,9% Sodium chloride (WVR, 27810.295P) solved in Milli-Q water), at 10^8^ and 10^10^ cells/mL, were used.

### 2.5. Bacteria-Specific Antibodies and Relevant Solutions

The polyclonal rabbit IgG (Thermo Fisher Scientific, Waltham, MA, USA, catalog number: PA1-7213), the polyclonal rabbit IgG FITC conjugate (Thermo Fisher Scientific, Waltham, MA, USA, catalog number: PA1-73029), the polyclonal goat IgG (Thermo Fisher Scientific, Waltham, MA, USA, catalog number: PA1-73032) against *E. coli* O/K serotype were used in our study. Additionally, monoclonal mouse anti *E. coli* IgG (Bio-Rad Laboratories Inc., Hercules, CA, USA, catalog number: OBT0749) and polyclonal rabbit anti *E. coli* IgG (Bio-Rad Laboratories Inc., Hercules, CA, USA, catalog number: 4329-4906) were purchased. The Bio-Rad OBT0749 does not cross-react with other members of the *Enterobacteriaceae*. The polyclonal rabbit anti-mouse IgG F(ab’)_2_ (Sigma-Aldrich Chemie GmbH, Darmstadt, Germany, catalog number: SAB3701000) was used as a negative control Ab. In the avidin–biotin-based immobilization experiments, biotinylated polyclonal rabbit IgG Ab (Thermo Fisher Scientific, Waltham, MA, USA, catalog number: PA1-25636) was employed. Biotinylation was performed with MxBIOS100-1KT Mix-n-stain biotin Ab labeling kit (Sigma-Aldrich Chemie GmbH, Darmstadt, Germany). The Abs were prepared in 25 mM MES (2-(N-morpholino)ethanesulfonic acid) buffer. The MES buffer was made from MES hydrate (Sigma-Aldrich Chemie GmbH, Darmstadt, Germany, catalog number: M2933) and the pH was adjusted to pH 6 with MES salt (Sigma-Aldrich Chemie GmbH, Darmstadt, Germany, catalog number: M3058). The concentration of the bulk Ab solution was 150 µg/mL, and the concentration of biotinylated Ab was 16 µg/mL.

### 2.6. Testing the Binding Ability of Anti-E. coli Antibodies Using ELISA

Recently several types of enzyme-linked immunosorbent assay (ELISA) techniques have been developed to obtain a reliable and measurable value about the specific antigen-Ab reaction. In our study, the binding ability of anti-*E. coli* Abs was determined with an in-house constructed sandwich ELISA. Different types of polyclonal Abs (Thermo Fisher PA1-73032 and PA1-7213, Bio-Rad 4329-4906) and a monoclonal Ab (Bio-Rad OBT0749) were tested. To prepare antigen-containing samples, *E. coli* DH5α strain was propagated on Mueller-Hinton agar plate (Bio-Rad Laboratories Inc., Hercules, CA, USA, catalog number: 64884) for 24 h. The colonies were collected with an inoculating loop and a bacterial suspension was prepared in a physiological salt solution to reach 4 McFarland density (approximately 1.2 × 10^9^ cells/mL). To measure the sensitivity, additional serial four-fold and ten-fold dilutions were made from the initial suspension. For ELISA measurement, 96 well plates (Thermo Fisher Scientific, Waltham, MA, USA, catalog number: 44-2404-21) with high protein binding capacity were applied. The plates were coated with 100 µL unconjugated Ab in 2 µg/mL concentration diluted in PBS overnight at room temperature. Plates were washed three times with 400 µL washing buffer containing 0.05% Tween 20 (VWR Chemicals, catalog number: 437082Q) in PBS and blocked with 300 µL 0.2% I-Block (Thermo Fisher Scientific, Waltham, MA, USA, catalog number: T2015) in PBS for 2 h. Plates were washed three times with washing buffer and 100 µL bacterium suspension was incubated for 2 h at room temperature. Following incubation, the plate was washed three times to remove unbound bacteria. Afterwards, 100 µL detection Ab in 100 ng/mL concentration was added to individual wells and incubated for 2 h allowing Abs to bind to the antigens. In the case of polyclonal Abs, the detection Ab was the same; however, it was biotinylated by a commercially available kit (Sigma-Aldrich Chemie GmbH, Darmstadt, Germany, catalog number: MXBIOS-100). In the case of monoclonal Ab (Bio-Rad OBT0749) for detection, a biotinylated Bio-Rad 4329-4906 polyclonal Ab was used. After washing the plate five times, 100 µL streptavidin-HRP (Thermo Fisher Scientific, Waltham, MA, USA, catalog number: 21130) was added in 1:20,000 dilution, and the solution was incubated for 30 min at room temperature. The plate was washed five times again, and 100 µL 3,3′,5,5′-tetramethylbenzidine (TMB) substrate (Thermo Fisher Scientific, Waltham, MA, USA, catalog number: SB02) was added, allowing for a 15 min incubation. The reaction was stopped with 100 µL 0.5 M sulfuric acid solution. Optical density was measured and assessed with an ELISA plate reader (Multiskan FC Microplate Photometer, Thermo Fisher Scientific, Waltham, MA, USA, catalog number: 51119000) at 450 nm with a reference filter of 620 nm.

## 3. Results and Discussion

### 3.1. Bacteria Repellent Coatings Tested by In Situ OWLS Measurements

**Figure 2:**
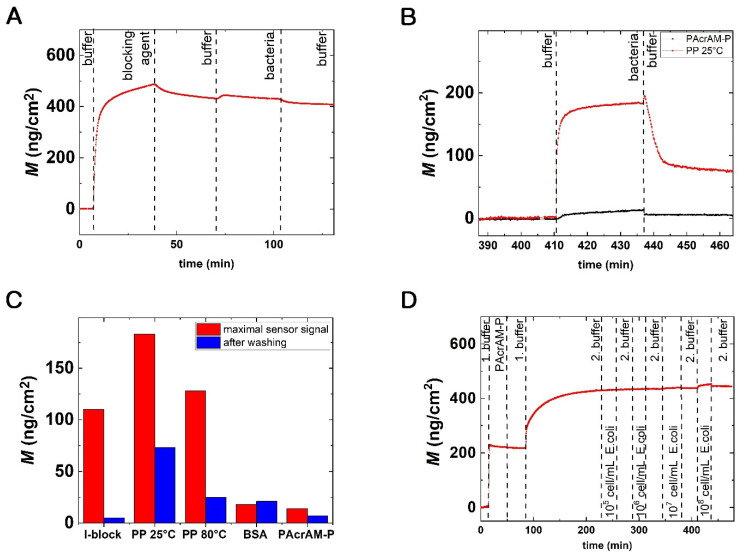
(**A**) Real-time measurement of bacterial adhesion on a bacteria repellent surface. A typical OWLS kinetic curve (the calculated surface adsorbed mass density, M, in real-time) is shown. In this specific case, the blocking agent was PAcrAM-P, showing excellent bacteria repulsion. (**B**) Comparing the OWLS signal of bacterial adhesion on PAcrAM-P and on PP (deposited at 25 °C) coated sensor surfaces. (**C**) The recorded maximum sensor signal after the addition of bacteria and the OWLS signal after washing off the irreversibly bound bacteria are shown for the various repellent coatings. (**D**) Real-time bacterial adhesion from four different bulk concentrations on the PAcrAM-P layer. 1. Buffer: 10 mM HEPES. 2. Buffer: PBS.

Altogether, five different strategies were investigated to create bacteria repellent coatings on OWLS sensor surfaces. The typical experimental curve with the measured surface mass densities is shown in Figure 2. First, the baseline was recorded in pure buffer (without bacteria) for approximately 10 min. After recording a stable baseline, the solution of the blocking agent was pumped into the OWLS cuvette. In all cases, the blocking solution was introduced for 30 min to adsorb as a monolayer on the OWLS chip surface. Afterward, the irreversibly bound blocking agent was removed by a 30 min washing step. Then a bacterial solution with 10^8^ cells/mL was pumped through the cuvette until 30 min had passed. Again, the loosely bound bacteria were washed off from the surface with pure buffer introduced for 30 min.

While the measured surface’s adsorbed mass from the OWLS measurement was precise for biomolecular layers, a calibration was needed to relate the surface adsorbed mass to the surface density of the adsorbed bacterial cells. From these calibration measurements using microscope images (for a typical image, see Supplementary Information (SI)), we determined that 100 adsorbed bacteria on a 1 mm^2^ area resulted in an OWLS signal of 3.48 ± 0.82 ng/cm^2^. This value is reasonable and results in the conclusion that the bottom surface of the bacterial mass (the volume inside the evanescent field) contained roughly 23 times more material than a compact protein layer. 

The measured bacteria repellent properties of the BSA, I-block, PP and PAcrAM-P coatings are highlighted in Figure 2B. Here, the recorded maximum sensor signal after bacterial addition and the OWLS signal after washing off the irreversibly bound bacteria are shown. Note, for the PP layer deposition, two coating temperatures were tested. The bacteria repellent capabilities of the formed layers were in increasing order: PP (with 25 °C coating temperature), I-block, PP (with 80 °C coating temperature), BSA and PAcrAM-P. 

Both BSA and I-block are considered efficient and widely used blocking agents [35,36,37,38,39]. In a study by Fu [40], evidence was found that I-block performs better than BSA. The surface was covered with 5% BSA to which more *Salmonella* bacteria could bind than to the I-block coated surface [40]. Our results support this finding, but only after washing off the loosely bound bacteria (see Figure 2C). Considering the data obtained for the PP films, our results are perfectly in line with previous literature. A temperature-induced, ultradense, PEG, polyelectrolyte surface grafting provided effective long-term bioresistance against mammalian cells, serum and whole blood [41]. In our experiments, PP layers deposited at high temperatures had roughly three times better bacteria repellent properties than the same layers deposited at room temperature. 

We obtained the best bacteria blocking results with the PAcrAM-P layers: the fewest cells could adhere on this surface, and half of the adhered bacteria could be washed off (see Figure 2B–D). Our results are in line with previous data on protein adsorption and mammalian cell adhesion. PAcrAm-P was originally developed to eliminate unwanted neuron cell–substrate interactions, although this new antifouling agent was previously used to repel mammalian cells and reduce protein surface adsorption. This polymer is composed of a poly(acrylamide) backbone, which adheres to the surface through electrostatic interactions, and the poly(2-methyl-2-oxazoline) (PMOXA) part repels the cells or proteins, which assures more effective and durable inhibition than that of PP [24]. 

The excellent bacteria repellent properties of the PAcrAM-P layer are further highlighted in Figure 2D. Here, a concentration series of a bacterial solution was injected onto the layer, and the OWLS signal was recorded in real-time. It is shown that at concentrations of 10^5^, 10^6^ and 10^7^ cells/mL the OWLS did not record any significant signal changes after bacteria injection. Within the resolution of the OWLS instrument, the layer was perfectly bacteria repellent. This result further verifies that 10^8^ cells/mL should be used for testing.

### 3.2. Binding Abilities of Different Antibodies Tested with ELISA

**Figure 3:**
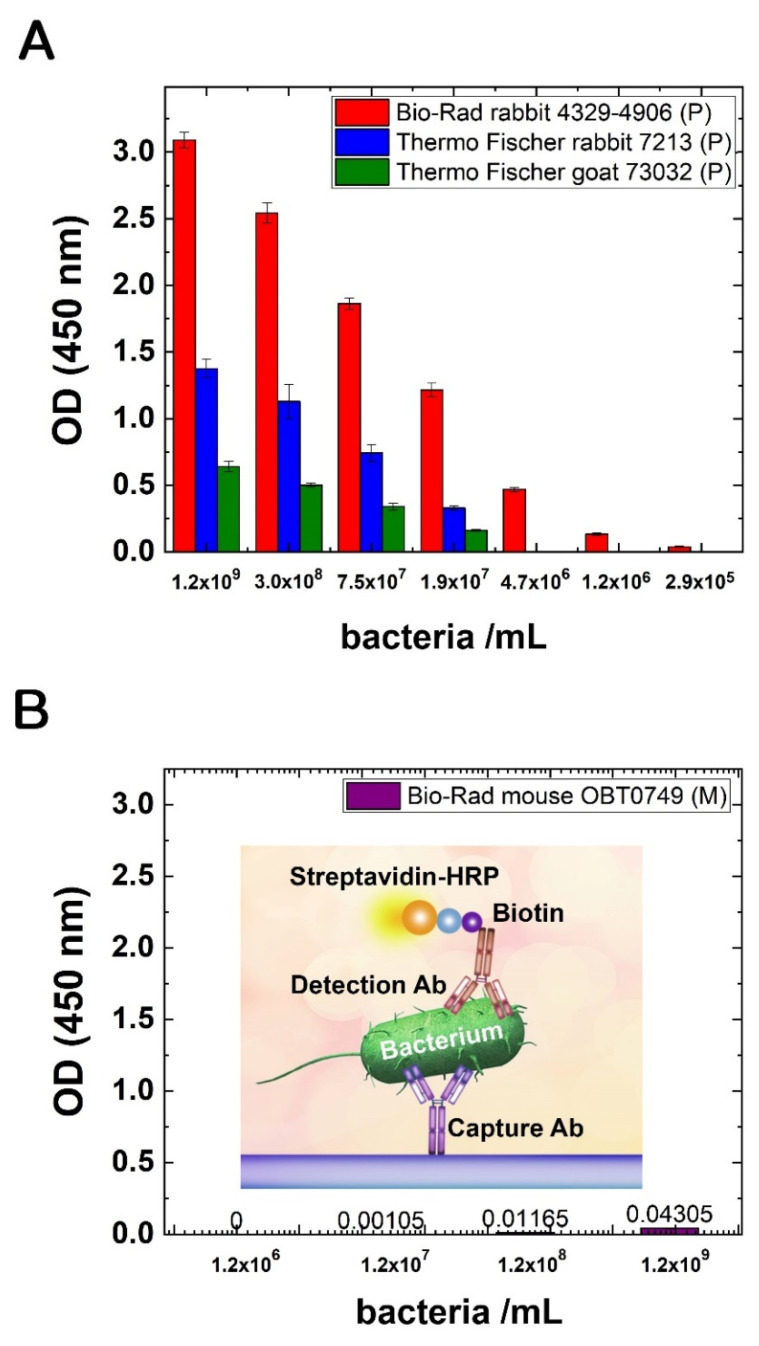
(**A**) Testing the binding abilities of different polyclonal Abs with an in-house ELISA test with different amounts of intact E. coli cells as antigen. Black: Bio-Rad 4329-4906, white: Thermo Fisher PA1-7213, grey: Thermo Fisher PA1-73032. (**B**) The binding ability of a monoclonal Ab (Bio-Rad OBT0749) with an in-house ELISA test with different amounts of intact *E. coli* cells as antigen. For detection, biotinylated polyclonal Ab (Bio-Rad 4329-4906) was used. The inset shows the schematic drawing of the assay.

All of the tested Abs could bind detectable amounts of *E. coli* cells; however, the ELISA assays showed different sensitivity. In general, the polyclonal Abs were performing better, and they could detect bacterial cells in smaller concentrations. Intact bacterial cells, as antigens, were used in a concentration range of approximately 3 × 10^5^–1.2 × 10^9^ cells/mL. In all cases of different Abs, the measured optical densities were decreasing in parallel with the descending amounts of antigen. The best performance was had by the Bio-Rad 4329-4906 polyclonal Ab with the lowest LOD: the signal was measurable even for approximately 1 × 10^6^ cells/mL. The other two polyclonal Abs (Thermo Fisher PA1-7213, PA1-73032) detected *E. coli* cells, although the lowest LOD was around 1.8 × 10^7^ cells/mL (Figure 3A). The affinity of the monoclonal Ab was the lowest compared to the others. The signal strength in ELISA was very low, even at the antigen concentration of 1.2 × 10^9^ cells/mL; in the lower range, no signal was detected. To detect bacteria in this concentration range, we used the best polyclonal Ab (Bio-Rad 4329-4906) as a receptor (detection) Ab (see Figure 3B); otherwise (with the other type of detection antibodies) no signal could be detected.

### 3.3. Various Strategies of Antibody Immobilization on OWLS Chip Surfaces

After the blocking experiments, we tested three different strategies to deposit the bacteria-specific Abs onto the OWLS chip surface. Namely, avidin-biotin type deposition of biotinylated Abs, Ab immobilization onto MG and protein A-based layers. The various steps of Ab immobilization were followed in real-time by the OWLS instrument, except in the case of protein A-type Ab immobilization. The activation of the chip surface and the covalent immobilization of protein A were performed ex situ, and only the specific binding of the Ab molecules was recorded in the OWLS instrument.

**Figure 4:**
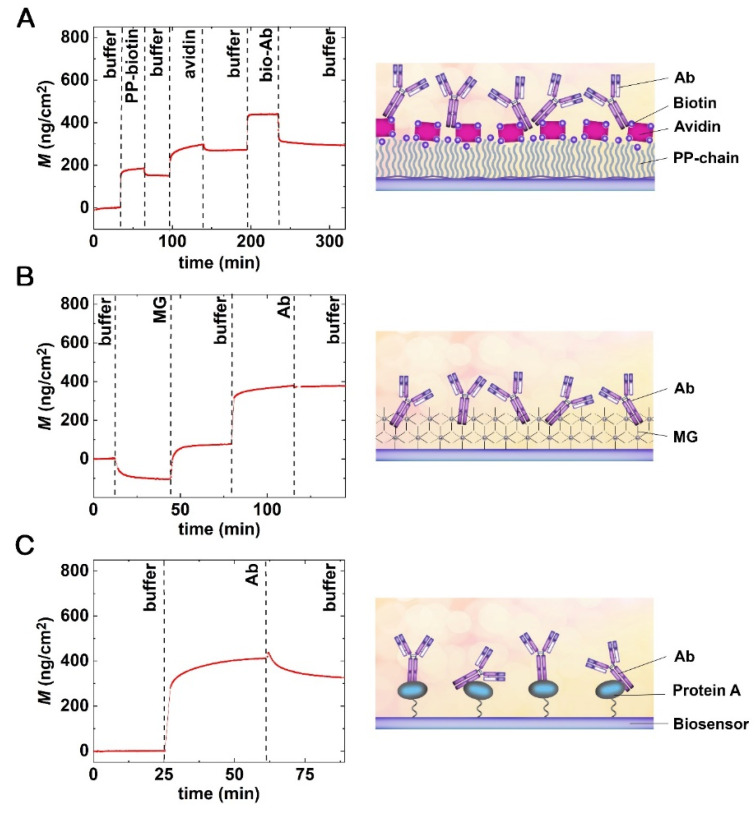
Real-time OWLS measurements of Ab immobilization and the schematic representations of the fabricated surface layers. (**A**) Avidin–biotin. (**B**) MG (now called AnteoBind). (**C**) Protein A-type immobilization of bacteria-specific Abs.

Figure 4A shows the real-time signal of layer deposition when biotinylated Abs were immobilized through the avidin–biotin linkage. The baseline was recorded in a buffer for 30 min, and PP-b was injected into the cuvette for 30 min. The amount of polymer deposited on the surface was approximately 200 ng/cm^2^. After the coating step, the excess polymer was washed off from the surface using a 30 min flow of buffer. In the next step, the avidin was injected into the cuvette for 30 min, which could specifically bind to the biotinylated surface, and again, the reversibly bound molecules were washed off for 30 min. The deposited avidin surface’s mass density was around 100 ng/cm^2^. Finally, the biotinylated Ab was immobilized on the avidin coated surface for 30 min, and the excess Abs were washed off by pure buffer. The immobilized Ab surface mass density was approximately 20 ng/cm^2^ in the case highlighted in Figure 4A. Note, this type of deposition is stable at pH 2–13, but reversible below pH 2 and above pH 13 [42].

Figure 4B depicts a typical real-time biosensor signal for Ab immobilization on an MG base layer. Again, the baseline was recorded in a buffer for 10 min; then, MG solution was injected into the cuvette for 30 min. Of note, the large negative signal was simply the consequence of the pH change (MG solutions are acidic with pH levels of 3.15–3.35), as the OWLS signal is pH sensitive. After the unbound immobilization reagents left the cuvette due to the buffer washing, the pH became stable again at 7.4. Afterward, the Ab solution was injected and left for 30 min. In the next step, the excess Ab was washed off the surface by pure buffer. Our results indicate that the MG polymer forms a nanometer-scale thin reactive layer and binds the Abs in a monolayer. 

The results of Ab deposition on a pre-deposited protein A layer are shown in Figure 4C. Here only the Ab deposition step is shown, as protein A covalent immobilization was performed outside of the OWLS cuvette (note, protein A immobilization was performed overnight on the GOPS covered chip’s surface). The OWLS measurement was started with a 10 min buffer baseline stabilization. After the stable baseline was reached, the Ab was captured by the protein A layer during a 30 min period. The excess Abs were washed off by pure buffer flow for 30 min. Our results indicate the successful formation of the stable Ab monolayer.

### 3.4. Surface Mass Densities and Structures of Antibody Layers

**Figure 5:**
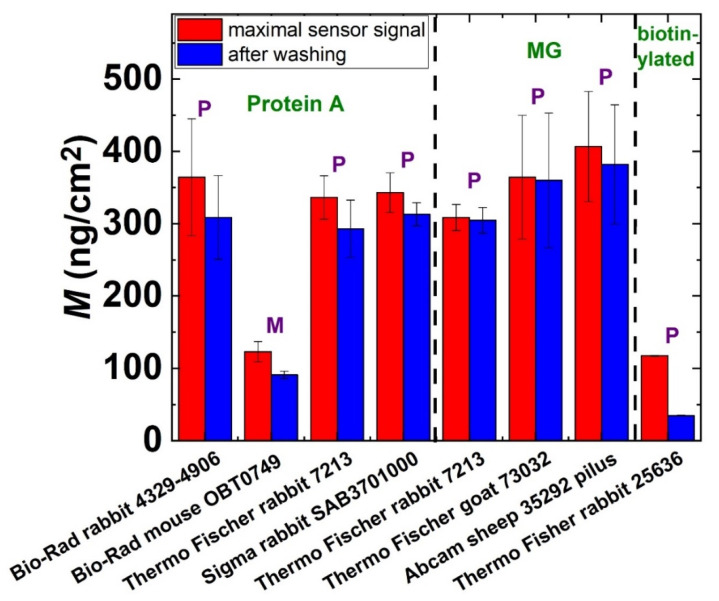
The measured surface mass densities of the Ab layers at the maximum sensor signal and after washing off the reversibly bound protein mass. Various immobilization strategies were employed and are marked in the figure. P: polyclonal, M: monoclonal.

The measured surface mass densities of the various Ab layers are summarized in Figure 5. In most cases, a dense Ab monolayer could be formed with surface mass densities in the range of 300–400 ng/cm^2^. However, our results clearly indicate that the IgG1 isotype monoclonal Ab binds much more weakly to the protein A layer than the polyclonal Abs and the polyclonal fragment (Sigma-Aldrich Chemie GmbH, Darmstadt, Germany, Rabbit, catalog number: SAB3701000). This low immobilization efficiency data could be explained by a weaker affinity of the Fc region to the protein A surface. It should be emphasized that the least immobilized Ab was obtained by the PP-b based avidin–biotin method, which resulted in an irreversibly bound Ab surface mass density of only 20 ng/cm^2^. We can also definitely declare that there were no significant differences between the MG and protein A immobilization strategies regarding the Ab amounts deposited in any repetition. Both methods are suitable for depositing a compact and stable Ab monolayer.

**Figure 6:**
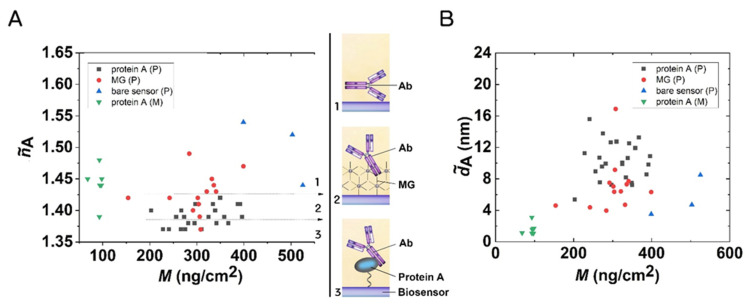
(**A**) Quasi-isotropic adlayer refractive index of the deposited Ab layers on MG, protein A, and bare chip surfaces. The shown values refer to the state when the excess was already washed off the surface. The magnitude of the refractive index indicates a possible orientation in the layer (see the drawings in the insets). (**B**) Quasi-homogeneous adlayer thickness of the Ab layers.

The structures of the formed layers are well characterized by the adlayer refractive index (${\tilde{n}}_{A}$ value) values calculated from the OWLS data using the homogeneous and isotropic thin adlayer model. This parameter can indicate the possible molecular orientation in the layer [43,44,45]. Figure 6 summarizes the results in the form of a scatter plot. The lowest ${\tilde{n}}_{A}$ value was obtained in the case of the protein A-based immobilization (~1.39), which suggests an end-on molecular orientation (see inset in Figure 6). A larger value (~1.43) was obtained in the case of MG, suggesting a more random layer. The large ${\tilde{n}}_{A}$ value on the bare sensor surfaces (~1.5) suggests positive birefringence, with a side-on molecular configuration [45].

The calculated ${\tilde{d}}_{A}$ values are plotted in Figure 6B. These data further confirm the above conclusions, suggesting the protein A-based Ab immobilization was mostly oriented layer towards the solution phase. 

### 3.5. Kinetic Analysis of Antibody Deposition—Kinetic Fits of Real-Time Biosensor Data

The measured kinetic OWLS data were fitted by a random sequential adsorption (RSA)-based parallel binding model supposing a reversibly and irreversibly bound molecular state with various footprints [45].

The corresponding equations are summarized below. The kinetic model describes binding events where a *P* protein molecule (Ab) can bind either reversibly or irreversibly to the surface resulting in bound states of *B*_r_ or *B*_i_, respectively. The schematic representation of the processes can be written as

(1) $$P\begin{array}{c} k_{a}\\ ⇌\\ k_{d} \end{array}B_{r};\;P\overset{k_{i}}{\to }B_{i}$$

where *k*_a_ and *k*_i_ are the reversible and irreversible association rate constants, and *k*_d_ is the dissociation rate constant in case of the reversible reaction. The kinetics of the above process can be described by Equations (2) and (3)—rate equations; and (4), the mass balance equation, as follows:

(2) $$dM_{r}\left(t\right)/dt=k_{a}c_{s}\phi \left(t\right)-k_{d}M_{r}\left(t\right)$$

(3) $$dM_{i}\left(t\right)/dt=k_{i}c_{s}\phi \left(t\right)$$

(4) $$M\left(t\right)=M_{r}\left(t\right)+M_{i}\left(t\right)$$

*M*_r_ and *M*_i_ are the mass of reversibly and irreversibly adsorbed Ab molecules deposited on the surface at time *t*; *M* is the total mass; *c*_s_ is the effective Ab concentration in the vicinity of the surface; *Φ* is the available surface function. *Φ* can be described by the fractional coverage *θ*. In the case of two separate footprints related to the two adsorbed states of the Ab, *θ* can be expressed as

(5) $$\Theta =1/\theta_{j}\left(M_{r}/\left(m/a_{r}\right)\right)+\left(M_{i}/\left(m/a_{i}\right)\right)$$

where *θ*_j_ is the jamming limit (model-limited level of adsorption), *m* is the mass of a single adsorbing Ab molecule (*m* = 2.5 × 10^−10^ ng, considering a molecular weight of 150 kDa) and *a*_r_ and *a*_i_ are the footprints (the area occupied by a surface-bound single Ab molecule) of the reversibly and irreversibly adsorbed molecules, respectively. According to the RSA model of adsorbing spherical objects, *Φ* is obtained as a polynomial function of *θ* [46]:

(6) $$\Phi ={\left(1-\theta \right)}^{3}/\left(1-0.812\;\cdot \;\theta -0.2336\;\cdot \;\theta^{2}+0.0845\;\cdot \;\theta^{3}\right)$$

The applied *θ*_j_ value corresponding to this RSA model was 0.547. Assuming that *c*_s_ is constant in time (d*c*_s_/d*t* = 0), one can calculate *c*_s_ using the following equation:

(7) $$c_{s}=\frac{c_{B}D/\delta_{D}+k_{d}M_{r}}{\left(k_{a}+k_{i}\right)\Phi +D/\delta_{D}}$$

where *D* is the diffusion coefficient of an Ab molecule (6.2 × 10^−13^ cm/s); *c*_B_ is the concentration of the bulk Ab solution (*c*_B_ = 150 µg/mL); *δ*_D_ is the thickness of the diffusion boundary layer, determined by the applied hydrodynamic conditions and calculated as

(8) $$\delta_{D}=\left(1/0.67\right){\left[\left(2/3\right)D\left(h/2\right)xA/Q\right]}^{1/3}$$

where *h*, *x* and *A* characterize the geometry of the OWLS flow cell (cell height *h* = 0.8 mm, distance from inlet *x* = 4 mm and cross-section *A* = 1.6 mm^2^), and *Q* is the volumetric flow rate (1 µL/s).

**Figure 7:**
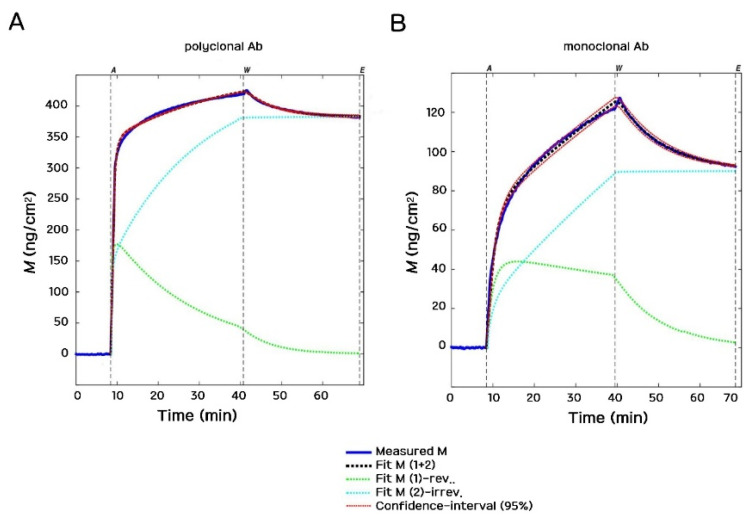
Typical OWLS kinetic curves of Ab deposition and their corresponding fits are shown. (**A**) Polyclonal Ab (Bio-Rad rabbit 4329-4906 on protein A surface). (**B**) Monoclonal Ab (Bio-Rad mouse OBT0749 on protein A surface). The smaller adsorbed surface mass density and slower adsorption are clearly visible in the case of the monoclonal Ab.

The model fits the total mass *M*(*t*) to the measured data while the association and dissociation rate constants of the Ab binding (*k*_a_, *k*_d_ and *k*_i_) and the molecular footprints of the bound Ab molecules (*a*_r_ and *a*_i_) are iterated as specified fit parameters. It can be seen in Figure 7 that the employed adsorption model fits our experimental data with excellent quality. Therein, typical examples of polyclonal and monoclonal Ab binding are shown. The much slower binding (and consequently the lower amount deposited) is clearly visible in the case of the monoclonal Ab.

**Figure 8:**
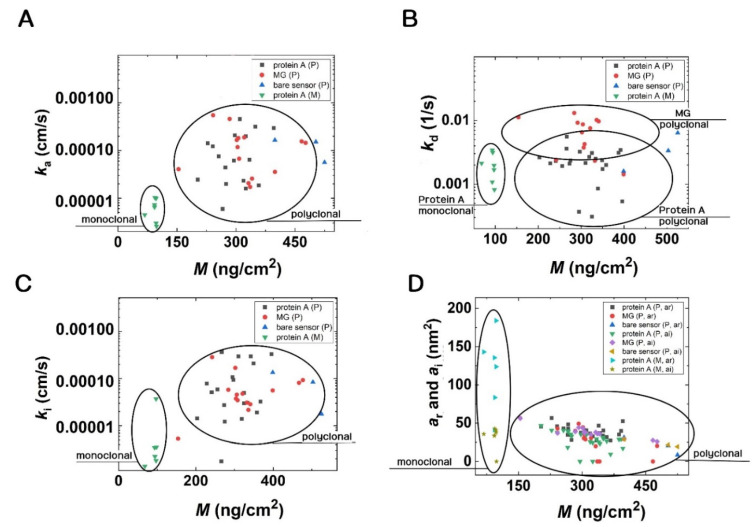
Scatter plots of data resulted from the kinetic fits. (**A**) The monoclonal and polyclonal Abs can be easily distinguished based on the adsorption rate constant (*k*_a_). (**B**) The different protein A and MG surfaces are very distinct, and additionally, the groups of polyclonal and monoclonal Abs are separated well from each other based on the dissociation rate constant (*k*_d_). (**C**) The polyclonal and monoclonal Abs groups are separated based on the irreversible association rate constants (*k*_i_). (**D**) Additionally, polyclonal and monoclonal Abs separate into different groups when the footprints of reversibly and irreversibly adsorbed molecules are considered.

The results of the kinetic fits are shown in Figure 8 in the form of scatter plots. (The obtained numerical values with errors are summarized in the SI, where the results in the form of box plots are also shown). The circles highlight the differences in Figure 8. First, the parameters are well separated for the monoclonal and polyclonal Abs. It is shown that the monoclonal Ab binding on protein A surfaces has slightly lower *k*_a_ and *k*_i_ values than those of the polyclonal Abs. These data points are well separated in Figure 8. The obtained standard errors are relatively large, but it is clear that Ab binding on the MG surface has a larger *k*_d_ value than that of protein A. Concerning the *k*_d_ data, a slightly larger value was obtained on the MG surfaces in the case of polyclonal Abs. Again, the mass vs. footprint data are well separated for the monoclonal and polyclonal Abs, but there are no significant differences between the values obtained at the various surfaces. Only a slightly decreasing footprint signal can be observed with increasing deposited mass. This is understandable and can be explained by molecular crowding.

We calculated the average values with the standard error for each parameter from the kinetic fits. First, we analyzed the polyclonal antibodies on different surfaces and did a one-way ANOVA to see if there were significant differences between the surfaces (see Table S1). We found that there were indeed significant differences in the *k*_d_ and *a*_r_ parameters. To further specify these differences, we performed post-hoc t-tests for these two parameters. The results are summarized in Table S2.

We were also interested in whether there was a difference between the kinetic parameters obtained with monoclonal and polyclonal antibody-coated surfaces. We had sufficient data for this analysis of protein A’s surface. (Note, we did not include the other two surfaces in this analysis because, as seen before, there were significant differences between the surfaces in the case of polyclonal antibodies (Table S2)). We performed one-way ANOVA on the data and found significant differences in the *k*_a_ and *a*_r_ parameters only (see Table S3).

### 3.6. Detection of Bacterial Adsorption on the Protein A-Based Antibody-Coated Surfaces with PAcrAM-P Blocking

Based on the above results, we chose the polyclonal Bio-Rad rabbit 4329-4906 Ab deposited on protein A surfaces and PAcrAM-P blocking for further experiments with bacteria-specific detection. This type of configuration of Ab immobilization and blocking resulted in the least nonspecifically bound amount of bacteria, and provided the largest immobilized Ab deposition densities with the preferred molecular orientation. 

**Figure 9:**
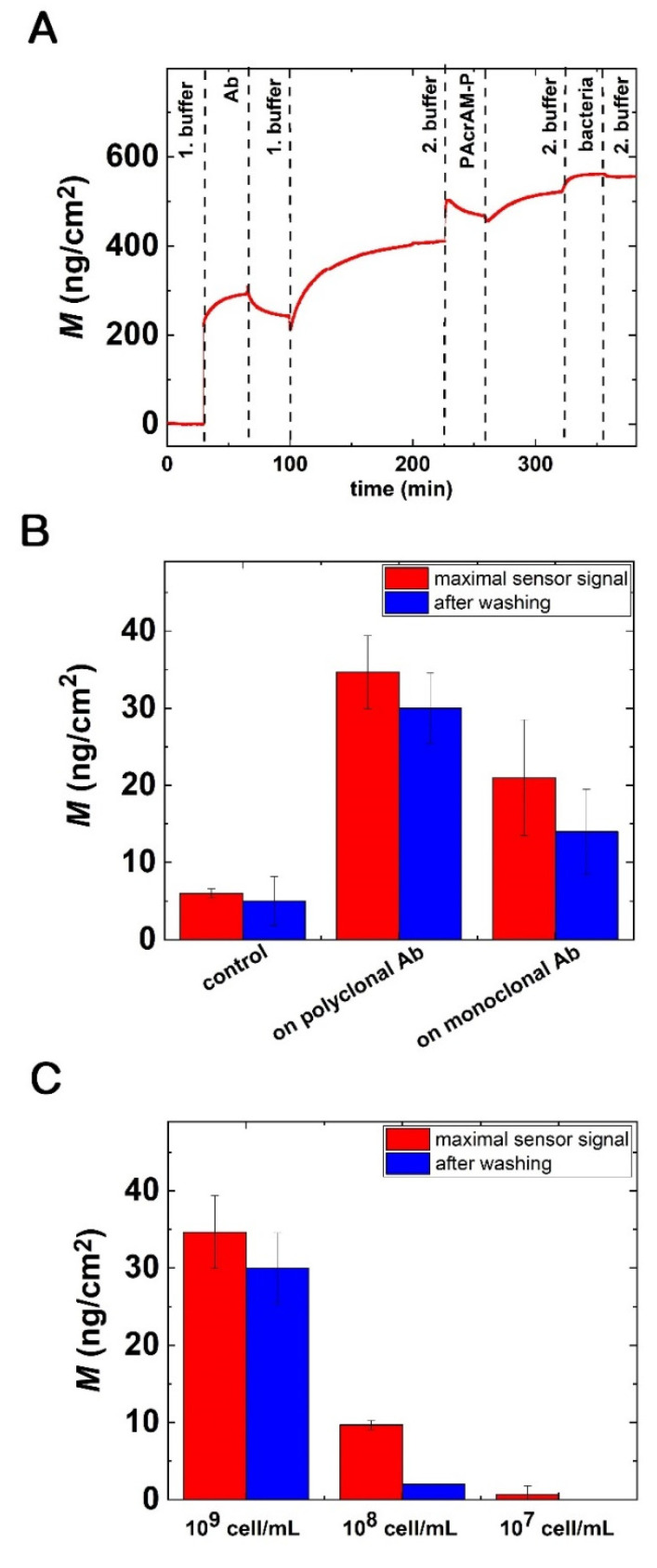
Bacterial adsorption on the Ab-coated sensor surfaces and the detection limit of OWLS. (**A**) Sensogram of a complete experiment, including the in situ coating procedures and subsequent bacteria adsorption for protein A-based immobilization and PAcrAM-P blocking. (**B**) *E. coli* adsorption on polyclonal Ab-coated surfaces using PAcrAM-P blocking; a 10^9^ cells/mL concentration was employed. The signals with statistics are clearly distinguishable from the relevant control signal (full layer without Ab). (**C**) OWLS signal for a series of bacterial concentrations using protein A-based immobilization with PAcrAM-P blocking.

Figure 9A highlights a typical kinetic OWLS curve, where the Ab deposition, blocking of remaining free areas with PAcrAM-P and subsequent bacterial adhesion are all shown. Such experiments were conducted without Ab immobilization (control surface) and while using the polyclonal (Bio-Rad rabbit 4329-4906) and monoclonal (Bio-Rad mouse OBT0749) Abs. Figure 9B highlights the results when the bacteria concentration was 10^9^ cells/mL. Of note, the recorded specific signals are well separated from the data of control measurements. Moreover, a larger specific signal was obtained for the polyclonal Ab, in full agreement with the ELISA data. 

In order to find the detection limit of the biosensor, bacteria concentrations with 10^8^ and 10^7^ cells/mL were also measured. The obtained results are summarized in Figure 9C. Of note, at these concentrations the PAcrAM-P reference surface provided perfect bacteria repellence within the resolution of the OWLS instrument. Therefore, one can conclude that the bulk detection limit of the present setup is around 10^7^ cells/mL.

In this case, it is important to note that the calculated bacteria surface density is around 70 bacteria/mm^2^. The above results indicate a considerable surface sensitivity but poor bacteria focusing and transfer efficiency towards the sensing layer. Note that this was not optimized in the present setup, which was mainly intended to develop the surface coatings. Another important parameter is the selectivity of the developed affinity layers. Although our main focus was to demonstrate the OWLS technique in the development of the coatings, we performed cross-reactivity experiments using a Gram-positive bacterium, *S. epidermidis*. Of note, negligible biosensor signal was obtained when employing the *E. coli*-specific antibody-coated surfaces and solutions of *S. epidermidis* (see Figure S4). 

As a future development, the measurement setup could be upgraded by an incorporated fluidic component that focuses bacteria close to the surface of the OWLS chip. There are several suitable sheath-flow-free microfluidic methods for particle and cell focusing which could be applicable for system integration [16]. Based on the utilized principle of operation, we can distinguish inertial focusing [47], such as lateral [48] or Dean-flow-coupled focusing [49], micro-thermal field-flow fractionation (Micro-TFFF) [50], isoelectric focusing [51], electrophoretic [52] and magnetophoretic focusing [53,54] deterministic lateral displacement [55] and different types of acoustic focusing, exploiting either surface acoustic waves (SAW) [56], bulk ultrasound standing waves (USW) [57] or acoustophoresis [58]. Out of these methods, acoustic focusing seems to be the most promising for our purposes, since it is label-free and can be implemented in situ with OWLS by integrating acoustic transducers and reflectors into the measurement chamber. Ultrasound standing waves were successfully integrated with label-free optical sensors (namely, a metal-clad leaky waveguide (MCLW) sensor) for bacteria focusing and detection [57]. In their work, Zourob et al. managed to focus 96% of bacterial spores from the bulk into the 1 µm vicinity of the sensor surface, from a solution of 1 × 10^6^ spores/mL [57]. For our system, which has a microfluidic chamber with a height of 800 µm, this focusing could mean a minimum of an 800× improvement in the LOD. While estimating the theoretical upper limit for a monolayer of bacteria on the sensor surface as 1 × 10^6^ bacteria/mm^2^ [57], and considering that our surface LOD is around 100 bacteria/mm^2^, the focusing could yield a maximum of 10,000× improvement in the LOD for bulk bacteria solutions.

## 4. Conclusions

The aim of the present study was to employ the OWLS technique in the development of bacteria repellent and bacteria adhesive antibody-coated surfaces. OWLS chips were coated with BSA, I-block, PAcrAM-P and PP (with different coating temperatures), and subsequent *E. coli* adhesion was monitored in situ. We found that the best performing blocking agents could inhibit bacterial adhesion from samples with bacteria concentrations of up to 10^7^ cells/mL. Note, in this study, only *E. coli*, a Gram-negative bacteria, was used to compare the performances of different repellent surfaces. However, based on our results, the OWLS technique is perfectly suitable for testing a wide range of bacterial species in a straightforward manner. Various immobilization methods were tested for depositing bacteria-specific antibodies on the biosensor’s surface. Simple physisorption, MG (AnteoBind) films, covalently immobilized protein A and avidin–biotin based surface chemistries were all fabricated and tested. The surface adsorbed mass densities of deposited antibodies were determined, and the OWLS kinetic data were evaluated to devise possible orientations of bacteria-capturing antibodies and to determine the rate constants and footprints of the binding events. The development of affinity layers was supported by ELISA measurements in order to test the bacteria binding capabilities of the antibodies. Our results have revealed some important differences between the MG (AnteoBind) and protein A-based Ab immobilization methods. Both methods are capable of depositing a stable and compact Ab layer, but the protein A-based method has a slightly better Ab orientation. This conclusion was obtained by comparing the quasi-homogeneous adlayer refractive index and thickness values measured for the two different layer types. Moreover, kinetic analysis of OWLS Ab deposition data showed a lower Ab desorption rate (*k*_d_) from the protein A-based layer, indicating stronger binding. This result is most probably the consequence of the two different Ab binding strategies, employing a specific binding pocket in the case of protein A, and multiple less specific, weaker bindings through metal chelate complexes in the case of MG. Another important difference is the time/effort required to prepare the coatings. In this regard, the simplicity and speed of the MG-based Ab immobilization clearly give it the advantage. These traits might be important in some specific real-life applications and compensate for its slightly lower performance. It is also important to note that the MG-based immobilization (AnteoBind) clearly outperformed the simple physisorption on the uncoated chip surfaces. The latter resulted in a completely wrong Ab orientation; Abs were deposited in a flat conformational state. Our current setup demonstrated a surface sensitivity of 70 bacteria/mm^2^. The present solution LOD could be significantly increased with future technical developments by incorporating bacteria focusing in the measurement setup.

## Data Availability

All data can be found in the manuscript.

## Supplementary Materials

The following supporting information can be downloaded at: https://www.mdpi.com/article/10.3390/bios12020056/s1, Figure S1: Typical microscope image of surface adsorbed bacteria on Ab (ThermoFischer goat 73032) coated sensor.; Table S1: Averages and standard error values of the obtained adsorption kinetic parameters for each surface with polyclonal antibodies.; Figure S2. The different kinetic parameters for each surface for the polyclonal antibodies are depicted with box plots. Figure S3. The different kinetic parameters for the polyclonal and monoclonal antibodies on protein A surface are depicted with box plots. Figure S4. Real-time OWLS measurements of S.epidermidis bacteria adhesion on E. coli specific antibody based surfaces. Table S2. The p values resulted from post-hoc t-test where the one-way ANOVA showed significant differences in Table S1. Table S3. Average and standard error values of the kinetic parameters for the polyclonal and monoclonal antibodies on Protein A surface and the p values resulted from one-way ANOVA.
