# Supplementary material for: Development and In-Depth Characterization of Bacteria Repellent and Bacteria Adhesive Antibody-Coated Surfaces Using Optical Waveguide Biosensing
Source: Biosensors (Basel). 2022 Jan 20;12(2):56. doi: 10.3390/bios12020056 (PMC8869200; doi:10.3390/bios12020056)
Supplement: Supplementary file 1 [file biosensors-12-00056-s001.zip › biosensors-1519019-supplementary.pdf]

*biosensors*

*Supplementary Materials*

# Development and In-Depth Characterization of Bacteria Repellent and Bacteria Adhesive Antibody-Coated Surfaces Using Optical Waveguide Biosensing

**Eniko Farkas [1], Robert Tarr [1,2], Tamás Gerecsei [1,3], Andras Saftics [1], Kinga Dóra Kovács [1,3], Balazs Stercz [4], Judit Domokos [4], Beatrix Peter [1], Sandor Kurunczi [1], Inna Szekacs [1], Attila Bonyár [2], Anita Bányai [5], Péter Fürjes [5], Szilvia Ruszkai-Szaniszló [6], Máté Varga [6], Barnabás Szabó [6], Eszter Ostorházi [4], Dóra Szabó [4] and Robert Horvath [1,*]**

[1] Centre for Energy Research, Nanobiosensorics Laboratory, Institute of Technical Physics and Materials Science, 1121, Budapest, Hungary; farkas.eniko@ek-cer.hu (E.F.); tarrr@edu.bme.hu (R.T.); tamas.gerecsei@cytosurge.com (T.G.); asaftics@coh.org (A.S.); kovacs.kinga.dora@energia.mta.hu (K.D.K.); peter.beatrix@energia.mta.hu (B.P.); kurunczi.sandor@energia.mta.hu (S.K.); szekacs.inna@ek-cer.hu (I.S.)
[2] Department of Electronics Technology, Faculty of Electrical Engineering and Informatics, Budapest University of Technology and Economics, 1111, Budapest, Hungary, bonyar@ett.bme.hu (A.B.)
[3] Department of Biological Physics, Eötvös Loránd University, 1117, Budapest, Hungary;
[4] Institute of Medical Microbiology, Semmelweis University, 1089, Budapest, Hungary; stercz.balazs@med.semmelweis-univ.hu (B.S.); domokos.judit@med.semmelweis-univ.hu (J.D.); ostorhazi.eszter@med.semmelweis-univ.hu (E.O.); szabo.dora@med.semmelweis-univ.hu (D.S.)
[5] Centre for Energy Research, Microsystems Lab, Institute of Technical Physics and Materials Science, 1121, Budapest, Hungary; banyai.anita@ek-cer.hu (A.B.); furjes.peter@energia.mta.hu (P.F.)
[6] 77 Elektronika Ltd., 1116 ,Budapest, Hungary; szszani@e77.hu (S.R.-S.); mvarga@e77.hu (M.V.); bszabo@e77.hu (B.S.)
* Correspondence: horvath.robert@energia.mta.hu (R.H.)

**Citation:** Farkas, E.; Tarr, R.; Gerecsei, T.; Saftics, A.; Kovács, K.D.; Stercz, B.; Domokos, J.; Peter, B.; Kurunczi, S.; Szekacs, I.; et al. Development and In-Depth Characterization of Bacteria Repel-Lent and Bacteria Adhesive Antibody-Coated Surfaces Using Opti-Cal Waveguide Biosensing. *Biosensors* **2022**, *12*, 56. https://doi.org/10.3390/bios12020056

Received: 5 December 2021
Accepted: 13 January 2022
Published: 20 January 2022

**Publisher's Note:** MDPI stays neutral with regard to jurisdictional claims in published maps and institutional affiliations.

**Copyright:** © 2022 by the authors. Licensee MDPI, Basel, Switzerland. This article is an open access article distributed under the terms and conditions of the Creative Commons Attribution (CC BY) license (https://creativecommons.org/licenses/by/4.0/).

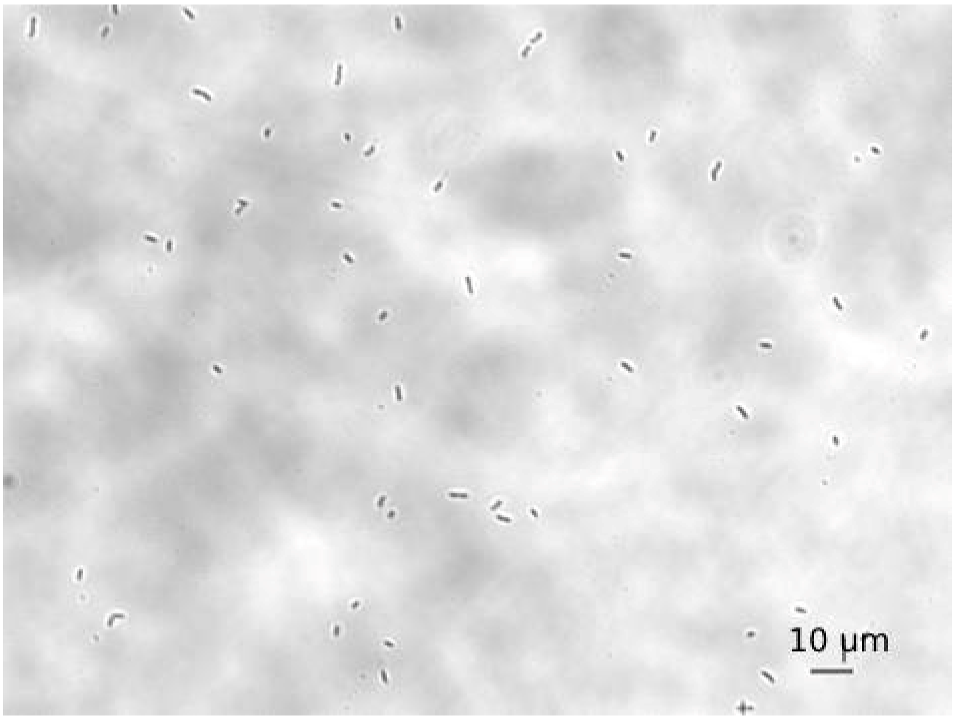


**Figure S1.** Typical microscope image of surface adsorbed bacteria on Ab (ThermoFischer goat 73032) coated sensor. Such images were taken of the OWLS chip surface after measurements, the number of bacteria was calculated for calibrating the recorded OWLS signal to the surface adsorbed density of bacteria (for results, see manuscript text in Results and Discussions).

**Table S1.** Averages and standard error values of the obtained adsorption kinetic parameters for each surface with polyclonal antibodies. The p values resulted from one-way ANOVA statistical analysis is also shown.

| | Polyclonal | | | | | | |
|---|---|---|---|---|---|---|---|
| | MG | Standard error | Protein A | Standard error | Bare sensor | Standard error | p |
| $k_a$ (cm/s) | 1.70E-04 | 4.80E-05 | 1.30E-04 | 3.20E-05 | 1.20E-04 | 3.40E-05 | 0.6 n.s. |
| $k_d$ (1/s) | 6.90E-03 | 1.10E-03 | 2.30E-03 | 2.50E-04 | 3.80E-03 | 1.40E-03 | 0.00003 **** |
| $k_i$ (cm/s) | 7.30E-05 | 2.80E-05 | 6.50E-04 | 3.80E-04 | 7.90E-05 | 3.40E-05 | 0.55 n.s. |
| $a_r$ ($nm^2$) | 23 | 5 | 39 | 1.6 | 20 | 6.5 | 0.00049 *** |
| $a_i$ ($nm^2$) | 36 | 1.5 | 28 | 2.4 | 23 | 2.9 | 0.07 n.s. |

****- significant level (p) less than 0.0001.
***- significant level (p) less than 0.001.

**Table S2.** The p values resulted from post-hoc t-test where the one-way ANOVA showed significant differences in Table S1.

| | MG vs. Protein A | MG vs. Bare sensor | Protein A vs. Bare sensor |
|---|---|---|---|
| $k_d$ | 0.000007 **** | 0.19 n.s. | 0.09 n.s. |
| $a_r$ | 0.00046 *** | 0.8 n.s. | 0.00099 *** |

****- significant level (p) less than 0.0001.
***- significant level (p) less than 0.001.

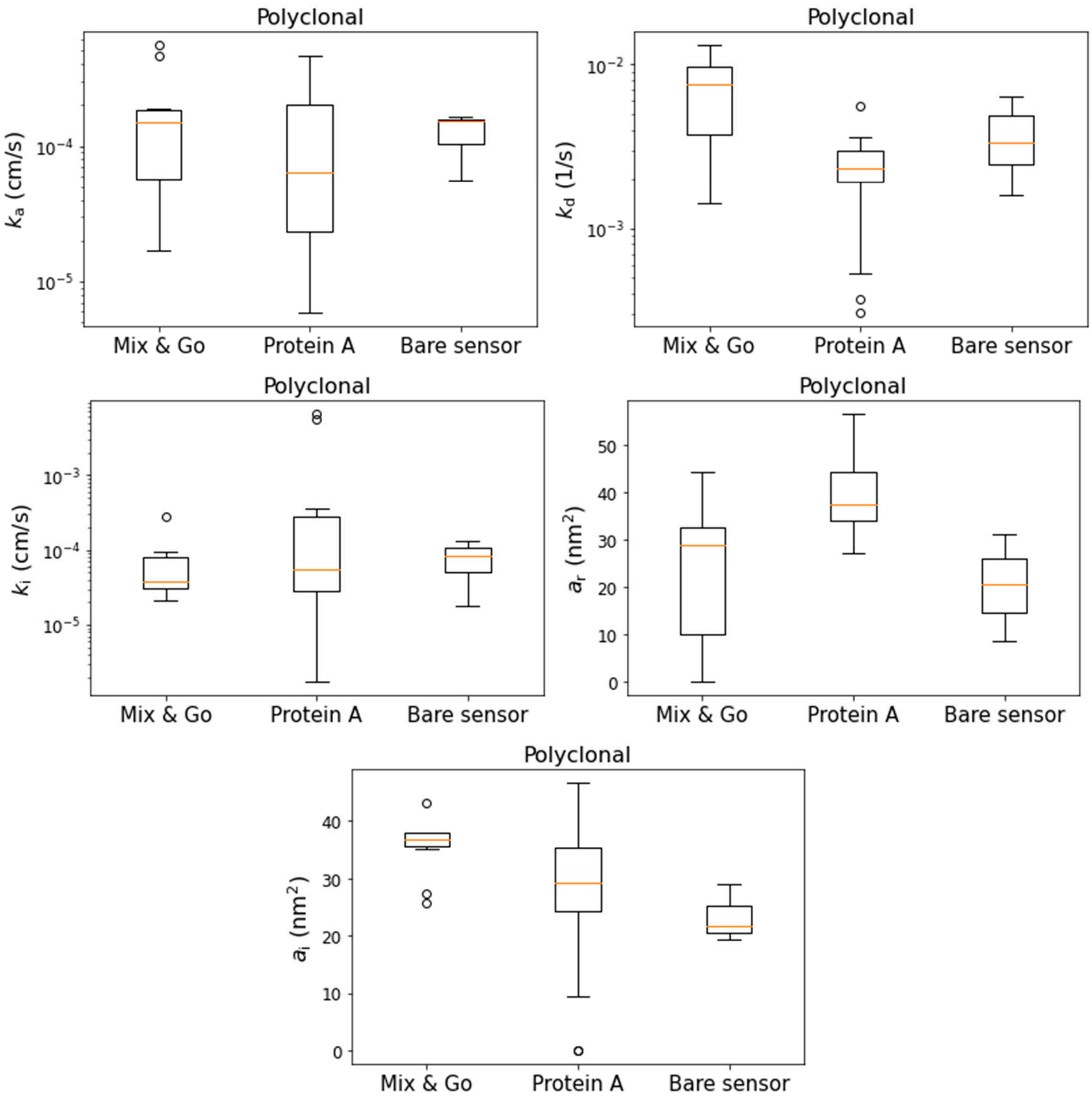


**Figure S2.** The different kinetic parameters for each surface for the polyclonal antibodies are depicted with box plots.

**Table S3.** Average and standard error values of the kinetic parameters for the polyclonal and monoclonal antibodies on Protein A surface and the p values resulted from one-way ANOVA.

| | Protein A | | | | |
|---|---|---|---|---|---|
| | Polyclonal | Standard error | Monoclonal | Standard error | p |
| **$k_a$ (cm/s)** | 1.30E-04 | 3.20E-05 | 6.30E-06 | 1.20E-06 | 0.03 * |
| **$k_d$ (1/s)** | 2.30E-03 | 2.50E-04 | 2.00E-03 | 3.70E-04 | 0.6 n.s. |
| **$k_i$ (cm/s)** | 6.50E-04 | 3.80E-04 | 7.20E-06 | 5.00E-06 | 0.4 n.s. |
| **$a_r$ (nm$^2$)** | 39 | 1.6 | 150 | 35 | 0.0000028 **** |
| **$a_i$ (nm$^2$)** | 28 | 2.4 | 32 | 5.5 | 0.4666 n.s. |

****- significant level (p) less than 0.0001.
*- significant level (p) less than 0.05.

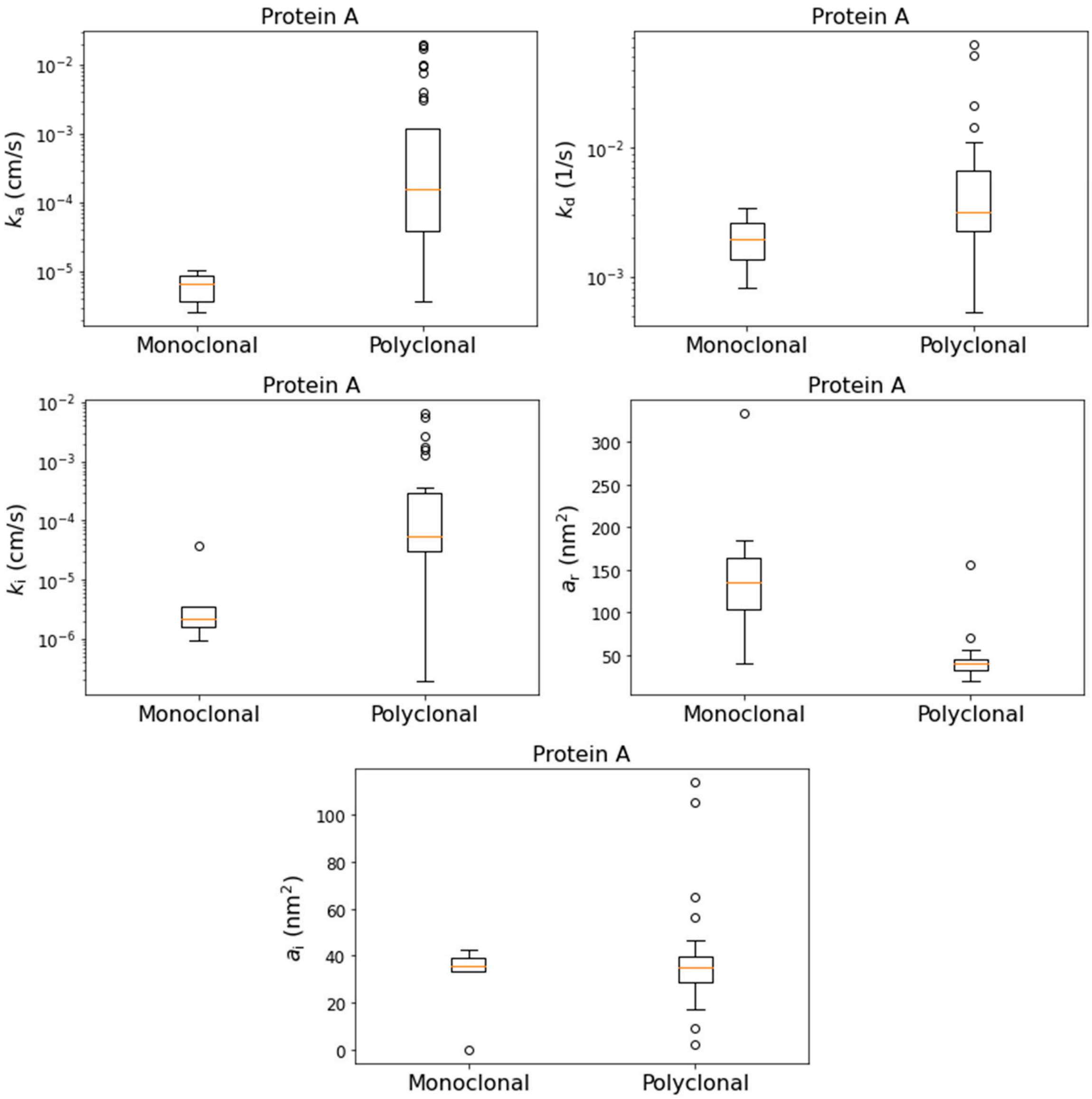


**Figure S3.** The different kinetic parameters for the polyclonal and monoclonal antibodies on protein A surface are depicted with box plots.

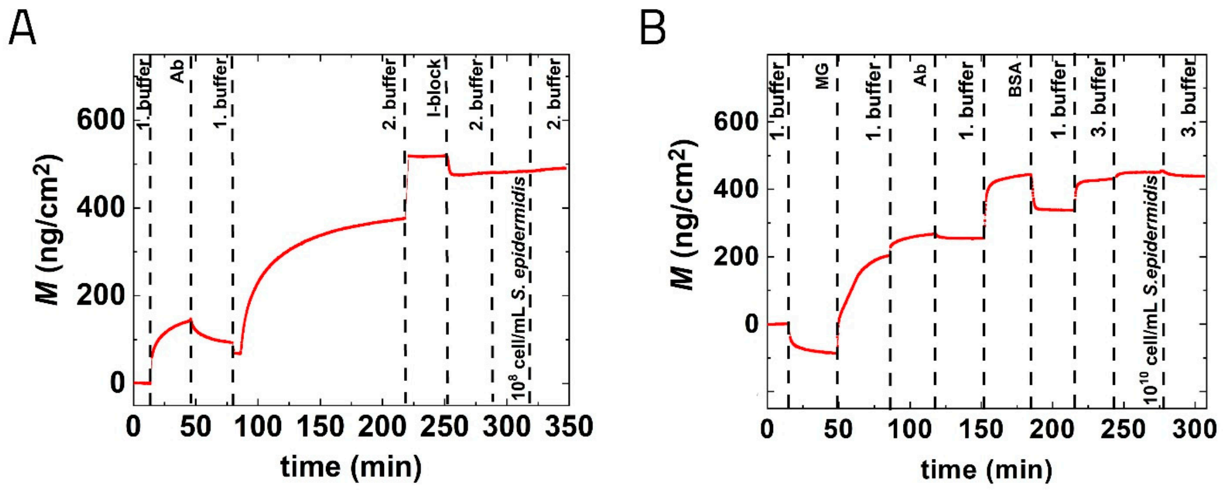


**Figure S4.** Real-time OWLS measurements of S.epidermidis bacteria adhesion on E. coli specific antibody based surfaces. (**A**) Sensogram of a complete experiment including the in situ coating procedures and subsequent bacteria ($10^8$ cell/mL) adsorption for protein A based immobilized Ab (Bio-Rad mouse OBT0749) with I-block blocking agent. **(B**) The complete OWLS measurement is shown Ab (Thermo Fisher PA1-7213) immobilization with MG and BSA blocking, followed by the adhesion of bacteria ($10^{10}$ cell/mL) on it. 1. buffer: MES buffer 2. buffer: PBS 3. buffer: physiological saline solution.
